# State of the art: noninvasive imaging and management of neurovascular trauma

**DOI:** 10.1186/1749-7922-2-1

**Published:** 2007-01-09

**Authors:** Charles E Ray, Shaun C Spalding, C Clay Cothren, Wei-Shin Wang, Ernest E Moore, Stephen P Johnson

**Affiliations:** 1 Diagnostic and Interventional Radiology, Denver Health Medical Center, 777 Bannock St, Denver, CO80204, USA; 2 Diagnostic and Interventional Radiology, University of Colorado Health Sciences Center, 4200 East Ninth Street, Denver, CO80220, USA; 3 Surgery, Denver Health Medical Center, 777 Bannock St., Denver, CO80204, USA; 4 Surgery, University of Colorado Health Sciences Center, 4200 East Ninth Street, Denver, CO80220, USA; 5 University of Colorado School of Medicine, 4200 East Ninth Street, Denver, CO80220, USA

## Abstract

Neurotrauma represents a significant public health problem, accounting for a significant proportion of the morbidity and mortality associated with all traumatic injuries. Both blunt and penetrating injuries to cervicocerebral vessels are significant and are likely more common than previously recognized. Imaging of such injuries is an important component in the evaluation of individuals presenting with such potential injuries, made all the more important since many of the vascular injuries are clinically silent. Management of injuries, particularly those caused by blunt trauma, is constantly evolving. This article addresses the current state of imaging and treatment of such injuries.

## Background

Trauma is the third leading cause of death in all age groups [1], and accounts for more deaths in children and young adults than any other etiology. In the United States, there are approximately 150,000 trauma-related deaths annually with 80% of these deaths occurring in the teenage population [2]. Trauma is also the major cause of death in men under the age of 35 years; more than 70% of these mortalities are directly related to neurotrauma [3].

Neurotrauma carries the greatest impact regarding death and functional impairment compared to all other types of trauma. In a study of deaths in road traffic fatalities, 79% of motor vehicle induced fatalities involved head injuries [4]. In an epidemiological study performed in Southern Australia, cranio-cerebral and spinal cord injuries were the leading cause of death in those up to age 49 years. With 71% of deaths in this study due to neurotrauma, it is evident that severe trauma to the head and neck account for disproportionate mortality, especially as it pertains to the disruption of the underlying neurovasculature [5,6].

Neurovascular trauma can be organized into two major categories by its mechanism: blunt and penetrating trauma. Each accounts for significant morbidity and mortality. One retrospective evaluation concluded that the morbidity and mortality were greater in those patients with penetrating carotid injuries than with blunt injuries, but that this was true only in those patients with multi-level penetrating injuries [7]. In addition, Landreneau et al. found that patients with penetrating injuries involving both the carotid and vertebral arteries were subject to 50% mortality as compared to 10% mortality from penetrating trauma to either vessel alone [8].

In contrast, blunt trauma may now surpass penetrating trauma in morbidity and mortality. Shackford, et al. reviewed trauma deaths in San Diego County and concluded that the most common mechanism of death was blunt trauma from motor vehicle collisions. This study also reported that the cause of death in nearly half of the trauma patients was due to central nervous system injuries [9]. Along with an increasing incidence of blunt trauma, a concomitant increase in the frequency of neurovascular injuries has been described. Blunt trauma is responsible for approximately 10% of all cervical vessel injuries, but the associated death and neurological dysfunction have been reported to be as high as 80% in those suffering from blunt carotid injuries [10]. Blunt vertebral arterial injuries, once thought to be innocuous, have been attributed to 24% of posterior circulation strokes and 8% of deaths in one study [11].

In this review, we will present and discuss blunt and penetrating trauma with concentration on their impact on the vasculature of the head and neck. The types of neurovascular injuries as a result of blunt and penetrating trauma will be introduced with the primary focus being on the imaging modalities and their findings. In addition, the role that neuroradiological intervention has in treatment of such injuries will be discussed.

## Anatomy

Neurovascular anatomy can be organized into the major vessels located in the cervical region and those vessels found in the cranium. The cervical region can also be subdivided into three zones that aid in the diagnosis and prognosis of neck trauma. The larger arteries and veins will be emphasized because they possess a greater potential for morbidity and mortality when they are injured; the larger vessels have also been more extensively studied in the literature. Along with outlining normal vascular anatomy, this section will introduce pertinent variant vascular anatomy in the neck and head, and address the significance that these variants have with regards to trauma and diagnostic imaging.

To appropriately evaluate neck injuries, the anterior neck is divided into three zones. The three zones are used in cases of penetrating trauma and provide prognostic values on the mortality of the patient [1]. The zones of the anterior neck also allow the caregiver to prioritize diagnostic and treatment options based on the location of the penetrating injury. Zone I is defined as extending inferiorly from the head of the clavicles, or from the cricoid cartilage, to the sternal notch [12,13]. A point to remember is that for Zone I, when the neck is in a neutral position, the cricoid cartilage is at the same level as the heads of the clavicles. When the head is extended, the cricoid cartilage is superior to the clavicular heads and expands Zone I slightly [1]. Injuries to Zone I have a high mortality rate, especially if penetrating trauma involves the vasculature or other structures within the thorax. Zone II encompasses the region from the top of Zone I to the angle of the mandible. Trauma to Zone II is clinically more apparent and surgical exposure is easier than in Zones I and III. Zone III covers the remaining anterior neck from the angle of the mandible to the base of the skull. Diagnostic studies are helpful and often required to assess injuries in Zones I and III due to the difficulty in obtaining surgical access in this area, although some investigators now question the utility of routine angiography for these injuries [14,15].

The cervical region contains, for our purposes here, two major paired arteries and their associated veins. The two major arteries are the vertebral arteries and the common carotid arteries. The vertebral arteries originate from the first portion of the subclavian arteries and travel anteriorly to the transverse processes of C7 before ascending vertically through the transverse foramina of the remaining cervical vertebrae. After passing through the foramen transversarium of C1, the vertebral arteries angle posteriorly almost at a right angle to their previous vertical path. The vertebral arteries lie on the bony lateral masses of the atlas just adjacent to the vertebral foramen and spinal cord. From the lateral masses of C1, the vertebral arteries penetrate the posterior atlanto-occipital membrane, dura, and arachnoid membranes to enter the subarachnoid space of the cerebellomedullary cistern. From the level of the foramen magnum, the vertebral arteries course anteriorly along the lateral surface of the medulla to unite on the caudal surface of the pons, forming the basilar artery [16].

The common carotid arteries originate within the thorax, the right common carotid stemming from the brachiocephalic trunk and the left common carotid arising directly from the arch of the aorta. The internal and external carotid arteries arise from the bifurcation of the common carotids, which are approximately at the level of the upper border of the thyroid cartilage and the level of C4. The external carotid arteries ascend anterosuperiorly and deep to the neck of the mandible. Branches include the superior thyroid, ascending pharyngeal, lingual, facial, occipital, posterior auricular, and superficial temporal arteries. It terminates in the maxillary artery [16].

The internal carotid artery ascends vertically, almost as an extension of the common carotid artery, to enter the carotid canal at the base of the skull. The internal carotid arteries have no branches in the cervical region. From the carotid canal, defined by the petrous portion of the temporal bone, the internal carotid artery enters the middle cranial fossa through the foramen lacerum. The internal carotid arteries angle anteriorly as they course within the cavernous sinus.

**Figure 1:**
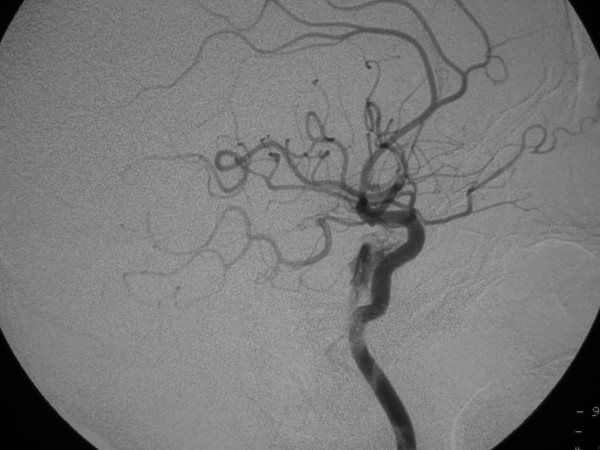
53-year old Korean man with a remote history of facial trauma, and a four day history of diplopia and right third cranial nerve palsy. Lateral digital subtraction angiogram, right internal carotid artery. During this early phase, there is visualization of the cervical and intracranial carotid artery; in addition, early filling of the cavernous sinus (arrow) is noted.

**Figure 2:**
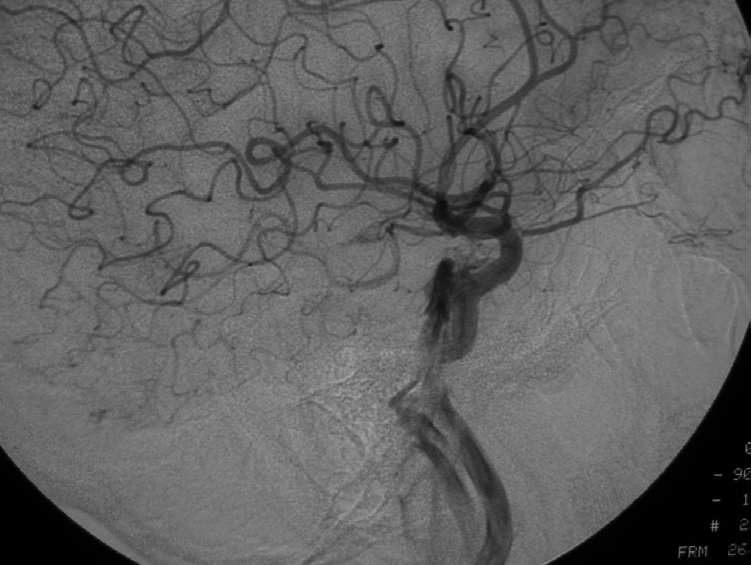
53-year old Korean man with a remote history of facial trauma, and a four day history of diplopia and right third cranial nerve palsy. Lateral digital subtraction angiogram, right internal carotid artery. Later image from the same angiogram as Figure 1A, demonstrating further filling of the cavernous sinus and draining veins.

**Figure 3:**
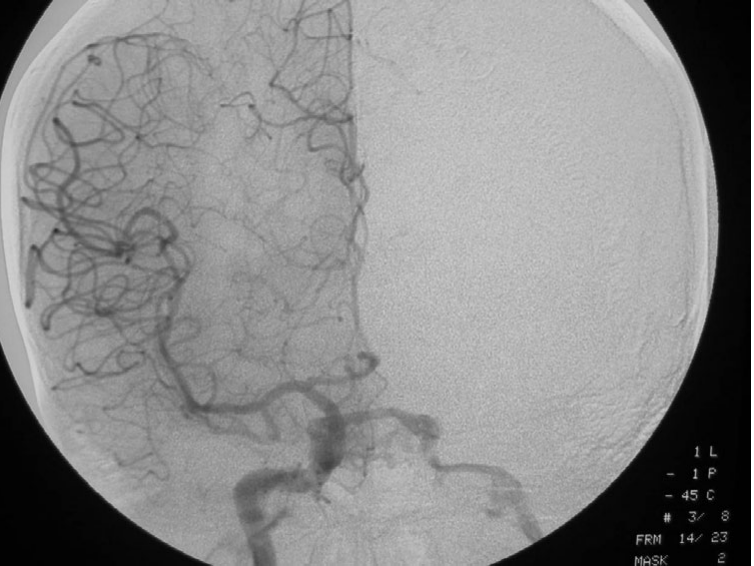
53-year old Korean man with a remote history of facial trauma, and a four day history of diplopia and right third cranial nerve palsy. Anteroposterior digital subtraction angiogram, right internal carotid artery. Mid-arterial phase image demonstrates early filling of the cavernous sinus with filling of the contralateral inferior petrosal sinus (arrow).

The cavernous sinus is an important structure to define due to its anatomic relationship to the internal carotid artery. The cavernous sinuses are part of the venous drainage of the head and are located on each side of the sella turcica. The two sinuses communicate with each other via intercavernous sinuses passing around the stalk of the pituitary gland. The significance of the cavernous sinus is due to its contents. The cavernous sinuses are a network of trabeculated veins whose endothelium encompasses cranial nerves III, IV, V1, V2, and VI. The intracavernous portion of the internal carotid arteries becomes completely surrounded by the venous plexus of the cavernous sinuses; the endothelium of the sinuses is the only layer separating the venous networks from the internal carotid arteries [16]. Head injuries that are severe enough to disrupt the integrity of the endothelium and the arterial wall can lead to carotid-cavernous sinus arteriovenous fistulas (CCF). Patients with CCF may complain of a self-audible bruit, proptosis, and visual impairment since the fistula impedes venous drainage from the ophthalmic veins. Angiographic findings include the early filling of the cavernous sinuses and the venous systems that receive their flow (Figures 1, 2, 3) [3].

From the cavernous sinuses, the internal carotid arteries ascend medially to the anterior clinoid process and exit the cavernous sinus to enter the subarachnoid space. The ophthalmic arteries are the first branch of the internal carotid arteries before they course inferior to the optic nerves and then laterally to the optic chiasm. From their position lateral to the optic chiasm, the internal carotid arteries ascend superiorly, terminating in the middle and anterior cerebral arteries at the level of the medial lateral sulcus. At the base of the brain, several important anastomoses between the branches of the internal carotid arteries and the basilar system help to form the cerebral arterial circle of Willis [16].

The circle of Willis, located in the interpeduncular fossa, is a network of arteries that joins the left and right internal carotid arteries with the basilar artery. The basilar artery gives several branches to the cerebellum before forming the left and right posterior cerebral arteries and the beginning of the circle of Willis. The posterior cerebral arteries anastomose with the internal carotid arteries by way of the paired posterior communicating arteries. To complete the circle, the anterior communicating artery joins the left and right anterior cerebral arteries [17]. The benefit of such an interconnection of vessels is to ensure continued blood supply to vital nervous tissue should one of the cerebral arteries become occluded.

Drainage of blood from the cranium is the role of the venous sinuses of the dura mater. The venous sinuses are conduits lacking valves, located between the dura mater and the internal periosteal lining of the skull. The sinuses receive blood from the cerebral veins. The most superiorly located of the venous sinuses is the superior sagittal sinus. The superior sagittal sinus extends from the crista galli and ends at the internal occipital protuberance in a dilation known as the confluence of the sinuses (torcula). Located along the posterior two-thirds of the inferior edge of the falx cerebri is the inferior sagittal sinus. This sinus receives blood from the cortical veins of the medial cerebral hemispheres. The inferior sagittal sinus ends by joining with the great cerebral vein of Galen, forming the straight sinus. The straight sinus travels along the falx cerebri where it is attached to the tentorium cerebelli in the posterior region of the cranium. The straight sinus drains predominantly into the left transverse sinus. The transverse sinuses originate at the confluence of the sinuses and travel laterally along the occipital bones and the posteroinferior angles of the parietal bones. Once the transverse sinuses exit the tentorium, they become the sigmoid sinuses. The sigmoid sinuses proceed anteriorly and laterally to the jugular tubercles of the occipital bones and terminate in the superior bulbs of the internal jugular veins [16].

### Variant vascular anatomy

Because of developmental variations, the normal vascular anatomy presented above can deviate from the norm and present diagnostic and therapeutic challenges following trauma to the head and neck. Aberrant vascular locations and failure of normal regression of embryological structures can complicate seemingly benign trauma by placing blood vessels in anatomic positions where they may be more easily injured. Although there are numerous anomalies, this chapter will focus on the arterial variants in three locations. These arterial variants include the aortic arch and its main branches in the chest, the vertebral and carotid arteries in the neck, and the circle of Willis in the head.

Classically, the aortic arch is found to be in the left mediastinum with three main branches stemming from its arch, but this anatomy occurs in only 70% of the population [18]. The most common anomaly of the aortic arch and its branches is a "bovine arch", representing a the left common carotid artery arising from the brachiocephalic artery rather than the aortic arch. This variant is found in 22% of people and accounts for 73% of all aortic arch branch irregularities [18]. Another less common anomaly of the aortic arch is the persistence of the fourth brachial arch, giving rise to a right-sided aortic arch. In this variant, the aortic arch and descending thoracic aorta exist in the right mediastinum and thorax, respectively. The right aortic arch is commonly associated with an aberrant left subclavian artery, which branches from the distal aortic arch and runs posteriorly in the chest to ultimately give rise to the left vertebral artery. A rare anomaly often associated with a right aortic arch is the cervical aortic arch. The cervical aortic arch, as the name implies, refers to a location of the aortic arch in the low or mid-neck. This higher than normal location of the aortic arch can jeopardize the integrity of the arch should an injury be sustained at the base of the neck. These aforementioned anomalies become important when evaluating penetrating trauma to the neck, especially those wounds involving Zone I of the anterior neck. Penetrating injuries to Zone I and the upper thorax may increase the likelihood of vascular damage if aberrant aortic arches and their branches are present. More importantly, knowledge of these anomalies becomes crucial in the evaluation of diagnostic imaging [17].

**Figure 4:**
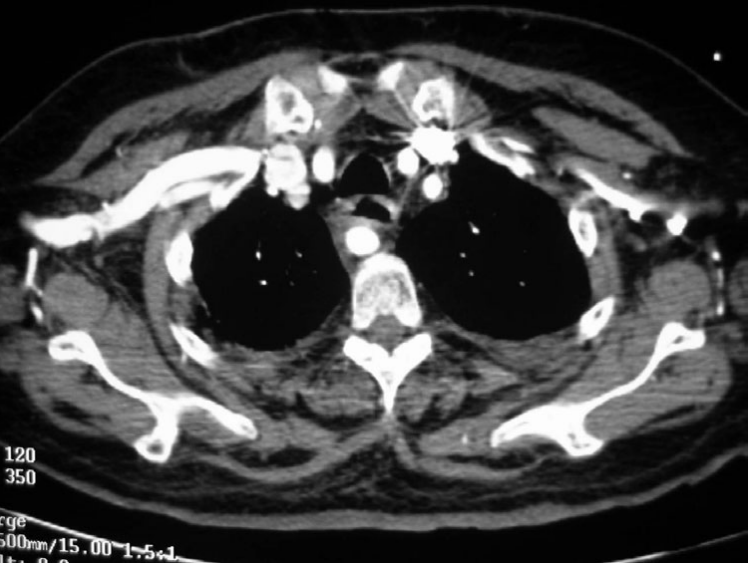
Anomalous origin on the right subclavian artery. Serial images from a helical CT scan of the superior mediastinum, cranial to caudal. Notice the large enhancing vascular structure posterior to the esophagus (*). On the more caudal images, a direct origin of this vessel from the aortic arch, distal to the origin of the left subclavian artery, is noted.

**Figure 5:**
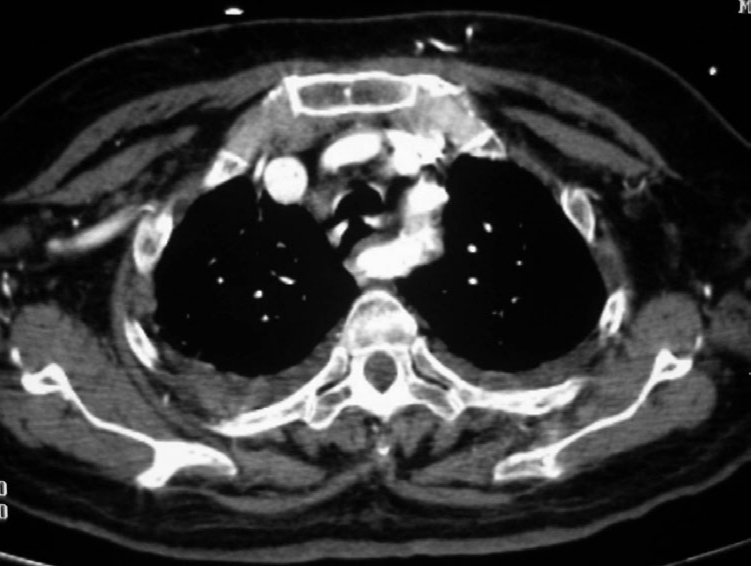
Anomalous origin on the right subclavian artery. Serial images from a helical CT scan of the superior mediastinum, cranial to caudal. Notice the large enhancing vascular structure posterior to the esophagus (*). On the more caudal images, a direct origin of this vessel from the aortic arch, distal to the origin of the left subclavian artery, is noted.

**Figure 6:**
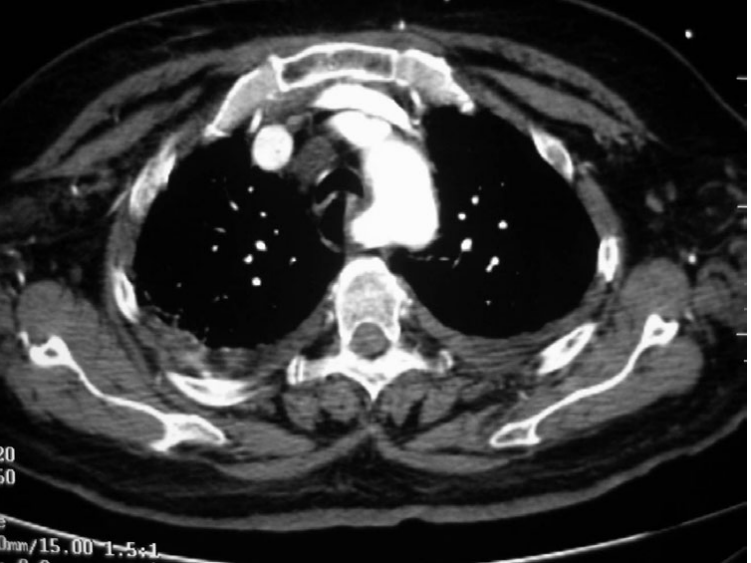
Anomalous origin on the right subclavian artery. Serial images from a helical CT scan of the superior mediastinum, cranial to caudal. Notice the large enhancing vascular structure posterior to the esophagus (*). On the more caudal images, a direct origin of this vessel from the aortic arch, distal to the origin of the left subclavian artery, is noted.

Another anomaly to be aware of is an aberrant right subclavian artery (Figures 4, 5, 6). Normally, the right subclavian artery is the continuation of the brachiocephalic trunk after the right common carotid artery origin. In 1% of the population, the right subclavian artery is the last branch of the aortic arch. From this distal position on the left aortic arch, the aberrant right subclavian artery travels posterior to the esophagus in 80% of patients, between the esophagus and trachea in 15%, and anterior to the trachea in the remaining 5% [18]. The presence of such an aberrant vessel and the course of the artery from the left thorax and neck to the right should be considered in injuries that involve trauma to the trachea and esophagus.

In 6% of the population, the left vertebral artery arises directly from the aortic arch, most commonly arising from the aortic arch between the left common carotid and the left subclavian arteries. In less than 1% of cases, the left vertebral artery is duplicated or has a bifid origin. In this case, both points of origin can be found on the aortic arch, or one point of origin is on the aortic arch and the other point from the left subclavian artery. In another variation, occuring in less than 1%, the left vertebral artery originates as the last branch of the aortic arch after the left subclavian [18]. Hypoplasia of one vertebral artery (usually the right) or both vertebral arteries may occur in cases involving the persistence of fetal carotid-vertebral arterial anastomoses [17].

Carotid arteries may also demonstrate variations. Bifurcation of the common carotid arteries can occur at any level between T2 in the chest and C1 in the neck. The external and internal carotid arteries may also originate separately and directly from the aortic arch. In these cases, the carotid arteries do not bifurcate or converge, and each artery traverses the length of the cervical area, spanning all three cervical zones. Finally, the right and left internal carotid arteries may form intercavernous anastomoses. This additional surface area of carotid artery within the cavernous sinuses theoretically increases the possibility of a carotid-cavernous fistula secondary to severe head trauma [17].

The circle of Willis has many variations, and in only 20% of cases does the arterial circle exist as a complete circle with equivalent flow throughout its circulation. The most common aberrancy is an absent or hypoplastic anterior cerebral artery (A1). In 20% of cases, the posterior communicating artery is absent or hypoplastic – this can occur either unilaterally or bilaterally [18]. The posterior cerebral circulation may arise from the internal carotid arteries rather than the vertebral arteries; in 20% of cases, the posterior cerebral arteries are branches of the internal carotid arteries. One or both vertebral arteries may terminate in the posterior inferior cerebellar artery before forming the basilar artery. In similar situations involving the fetal anatomy of the posterior circulation, the posterior communicating arteries are the major route for blood flow to the posterior cerebral arteries [17].

## Vascular trauma

Whether the mechanism of injury is due to blunt or penetrating trauma, the subsequent injury to the underlying vasculature can be categorized into five types based on their appearance on imaging studies. The five categories of vascular injury include: (1) intimal and/or medial damage with or without associated narrowing of the vessel lumen and/or dissection of the vessel wall, (2) aneurysm or pseudoaneurysm, (3) complete occlusion of the vessel lumen, (4) arteriovenous fistula, and (5) complete transection.

Significant morbidity and mortality are associated with trauma to the carotid arteries [7]. This is in part due to the fact that blunt trauma to the carotid arteries is usually not evident by physical exam as few external signs are present, and neurological symptoms are not always apparent at presentation [19,20]. Being cognizant of anatomical locations of the carotid arteries that are more susceptible to injury, as well as the injury types associated with specific mechanisms of trauma, can help reduce the morbidity and mortality associated with such trauma. One anatomical location associated with carotid artery injury in the neck is the close proximity of the carotid arteries to the lateral processes of the upper cervical vertebrae [19]. This location, along with severe hyperextension and abrupt flexion of the head, can lead to carotid artery dissection [21]. The disruption of the intima of the injured carotid artery has been postulated to occur due to the severe flexion or extension of the head causing the carotid artery to become trapped and compressed between the angle of the mandible and the lateral processes of the upper cervical vertebrae. This may be particularly evident if there is an associated rotational component to the injury. Dissection of the carotid artery has also been reported in cases of mandible fractures and can occur simultaneously in both carotid arteries following motor vehicle collisions [22,23]. In most carotid injuries, there exists some disruption of the intima that may potentially lead to neurological compromise. One theory is that the intimal disruption leads to superimposed platelet aggregation and thrombus formation, predisposing to embolism to the cerebral circulation. The intimal injury often occurs with associated medial injury that can cause dissection with significant luminal narrowing or even complete occlusion of the injured vessel [24].

**Figure 7:**
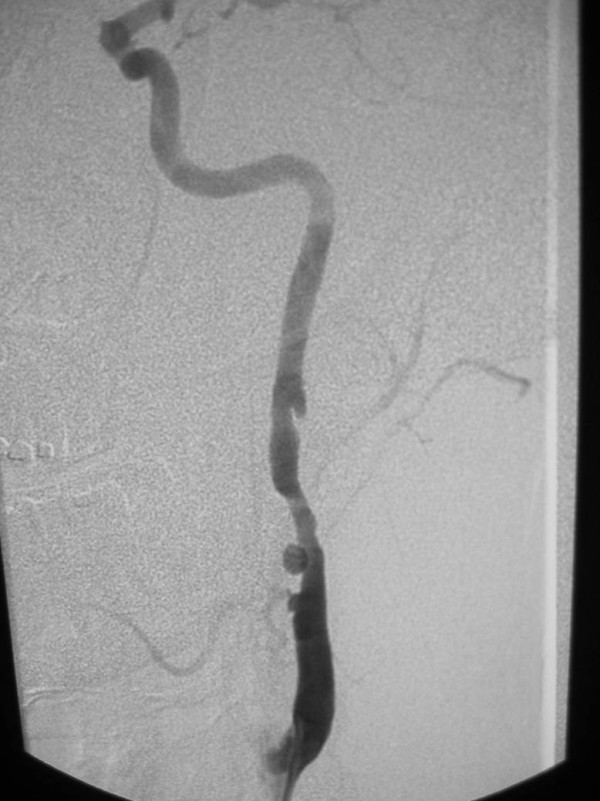
36-year old woman, s/p motor vehicle collision. On admission, an angiogram demonstrated a pseudoaneurysm of the thoracic aorta, as well as a grade 3 (pseudoaneurysm) of her left internal carotid artery. Her aorta was repaired immediately; on follow-up angiography of her carotid injury, her pseudoaneurysm had progressed and there was significant narrowing of the adjacent internal carotid artery. Digital subtraction angiogram of the left internal carotid artery, 7-days following initial injury. There is a pseudoaneurysm (arrow) of the proximal internal carotid artery with narrowing of the adjacent artery. A second, smaller pseudoaneurysm (arrowhead) is noted in the more distal artery.

**Figure 8:**
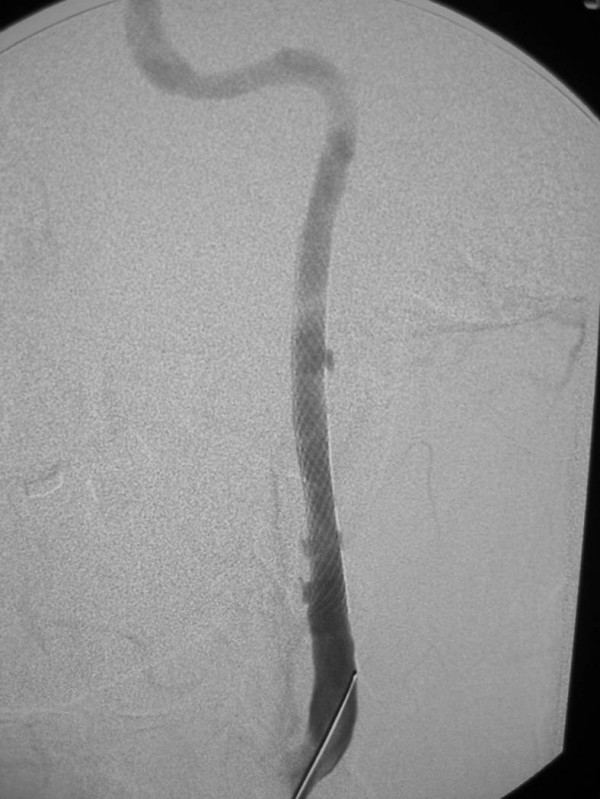
36-year old woman, s/p motor vehicle collision. On admission, an angiogram demonstrated a pseudoaneurysm of the thoracic aorta, as well as a grade 3 (pseudoaneurysm) of her left internal carotid artery. Her aorta was repaired immediately; on follow-up angiography of her carotid injury, her pseudoaneurysm had progressed and there was significant narrowing of the adjacent internal carotid artery. Digital subtraction angiogram of the left internal carotid artery, s/p stent placement. A 6 mm × 47 mm Magic Wallstent (Boston Scientific, Watertown, MA) was placed. Notice the immediate and nearly complete resolution of the pseudoaneurysms.

**Figure 9:**
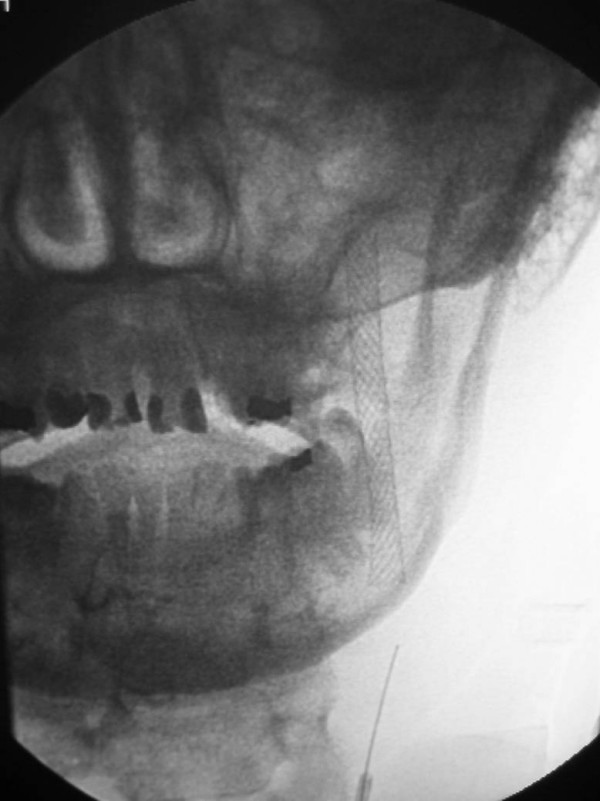
36-year old woman, s/p motor vehicle collision. On admission, an angiogram demonstrated a pseudoaneurysm of the thoracic aorta, as well as a grade 3 (pseudoaneurysm) of her left internal carotid artery. Her aorta was repaired immediately; on follow-up angiography of her carotid injury, her pseudoaneurysm had progressed and there was significant narrowing of the adjacent internal carotid artery. Unsubtracted image demonstrating the cervical stent (arrows).

Blunt cervicovascular trauma has increased in incidence, leading to increased morbidity [11,25-27]. Biffl et al proposed a grading scheme for blunt carotid artery injuries in an attempt to assess neurological outcome based on the mechanism of injury [28]. Grade I injuries are mild intimal injuries with luminal narrowing <25%. Grade II injuries are intimal injuries, dissections, orintramural hematomas with luminal stenosis >25%. Grade III injuries have pseudoaneurysm formation, grade IV injuries are vessel occlusions, and grade V injuries are complete transections (Table 1). In this initial publication, injuries of different grades had different outcomes. Two-thirds of mild intimal injuries (Grade I) healed regardless of treatment. Grade II injuries progressed despite heparin in 70% of cases. Only 8% of pseudoaneurysms (GradeIII) injuries healed with heparin therapy, but 70% healed after stent placement (Figures 7, 8, 9). Grade IV injuries did not recanalize spontaneously in the early post-operative period, and grade V injuries were usually lethal and refractory to treatment. Stroke risk increased with injury grade [28].

**Table 1:** Grading system for blunt cerebrovascular injuries

**Grade**	**Injury pattern**
I	Intimal injury, luminal narrowing <25%
II	Visualized dissection flap, or luminal narrowing >25%
III	Pseudoaneurysm
IV	Vessel occlusion
V	Vessel transection

Adapted from Reference 28.

Similar to blunt carotid trauma, vertebral artery injury can cause significant neurological compromise [11]. Trauma to the vertebral arteries can occur at several anatomical locations. As the vertebral arteries course through the cervical region they become more vulnerable where they course adjacent to more rigid structures. Muscular tissue and fibrous bands traversing the arteries' paths, prior to the vessels entering the foramen transversarium of C6, along with osteophytic spurs in the midcervical spine can act as fixed points against which the vertebral arteries can be damaged during blunt trauma [29]. The vertebral arteries can also be injured where they run in a fixed position within the transverse foramen of C1–6. Motion at the atlantoaxial joint, cervical fractures, and dislocations with bony impingements can all lead to intimal and medial injuries. These injuries can produce ischemia from vessel lumen narrowing or complete occlusion, or embolization of thrombus arising from pseudoaneurysms or dissection [30-32]. Neurological compromise can occur when there is insufficient contralateral circulation from the uninjured vertebral artery. If there are any vascular anomalies that prevent adequate contralateral circulation, then the morbidity and mortality from the injury can increase dramatically.

The intracranial arteries are also subject to injury from both blunt and penetrating trauma. Blunt trauma generally causes intimal damage that can manifest with dissection and extravasation as well as with the formation of intraluminal thromboses [33]. In the setting of blunt trauma, the internal carotid artery and becomes vulnerable where it is fixed in position by the surrounding petrous and cavernous portions of the skull [19]. Blunt head injuries can cause damage to the internal carotid arteries by producing shearing forces at the point where the arteries enter the skull, or by causing transection of the arteries from fractured bone fragments [34]. In cases without basal skull fractures, the internal carotid arteries are typically injured at the point where they exit the cavernous sinus. Penetrating trauma usually manifests itself in the injured carotid artery as an intimal tear with its sequelae of a pseudoaneurysm. Often the injuries affecting the internal carotid arteries at the base of the skull are located at the level of C1 in Zone III of the anterior neck [19].

Blunt or penetrating trauma to the head and face may produce a fistula anywhere from the cervical region to the cortical arterial branches. The most common trauma-induced intracranial arteriovenous fistula occurs between the internal carotid artery and the cavernous sinus. A carotid-cavernous fistula (CCF) can be clinically silent for days to weeks following the initial trauma [35]. The injury resulting in the fistula can range from an avulsion of a small cavernous branch to complete transection of the internal carotid artery. The carotid-cavernous fistula can also arise spontaneously as a result of a ruptured aneurysm.

Another relatively common vessel in the head to give rise to a post-traumatic arteriovenous fistula is the middle meningeal artery [36,37]. In one review of 446 trauma patients, the incidence of middle meningeal artery arteriovenous fistulas was 2%. The mortality rate associated with middle meningeal artery AVFs is high. Whether or not the high mortality rate is due to concomitant injuries is unknown, but the middle meningeal artery distribution should be thoroughly evaluated during angiography.

The middle cerebral artery may be injured following blunt trauma to the head or face and is a common site for arterial dissection. Sato et al reported that the middle cerebral artery had been injured in a large number of cases of head trauma, and that there was a predominance of right cerebral artery injuries associated with the head trauma [38]. The most common site of origin for dissection of the middle cerebral artery is at the proximal portion of the vessel with extension to part or all of the M1 segment. The importance of evaluating the middle cerebral artery is that the mortality associated with traumatic injury to this artery is very high, and the neurological complications can be severe.

In addition to arterial injury, the venous sinuses are also vulnerable to traumatic injury. The venous sinuses, particularly the superior sagittal, the transverse, and the sigmoid sinuses, are very susceptible to laceration and extravasation of large volumes of blood. Given the superficial anatomic location of the venous sinuses, penetrating trauma to the head as well as blunt trauma causing fractures can easily disrupt the integrity of the venous sinuses [19]. Most often, trauma causes tears in the venous sinuses and leads to extravasation of blood into the subdural or epidural spaces. It is not uncommon for trauma to create a venous sinus occlusion, which may increase the intracranial pressure and cause associated neurological symptoms [19].

## Treatment

Once a vascular injury is localized and identified, the appropriate treatment may be initiated. Treatment options depend on the risk-benefit ratio as it relates to the injury, the collateral circulation of the brain, and the presence of or potential for thrombosis of the injured vessel [39]. The most important variable directly affecting the risk-benefit ratio is the neurologic status of the patient [19]. In a study by Scalfani et al, of the four deaths encountered from penetrating neck trauma, three of the deaths were independent of the treatment option, but rather were associated with neurologic deficits on admission [40]. Also, McKevitt et al noted that a Glasgow Coma Scale of eight or less following blunt vascular neck injuries is an independent variable predicting stroke or death [41]. In a separate study performed of 24 patients with penetrating trauma to the cervical carotid arteries, the outcome of patients with preoperative neurological deficits was unaffected by either repair or ligation [42].

When the injured vessel may require ligation, embolization, or even repair with temporary cessation of blood flow, the collateral circulation of the brain becomes important. Pre-intervention assessment of the contralateral circulation can be performed during diagnostic imaging. A balloon can be inflated in the proximal portion of the injured vessel and, if the patient's clinical status allows, neurologic performance can be evaluated [43]. If there exists a new neurologic deficit with temporary balloon occlusion, the collateral circulation is inadequate to allow treatment options that will significantly decrease blood flow.

The literature is replete with varying treatment modalities for the numerous different vascular injuries encountered. The treatment options for neurovascular trauma range from simply observing the patient to surgical and endovascular repair [44]. The various treatment methods can be categorized into three groups based on the degree of invasiveness. The three treatment generally discussed groups are observation/medical therapy, surgical repair/ligation of the injured vessels, and transcatheter treatment, including embolization and stents.

### Medical therapy

The first treatment group includes observation with serial imaging and anticoagulation therapy. When angiography reveals a vessel injury, observation as a treatment option may be considered depending on the injury grade, when access to the injured area is problematic, and the patient is neurologically stable. In a study by Richardson et al, four patients with injuries to the distal carotid arteries were observed with serial angiography [45]. The portion of the internal carotid artery that was injured proved to be difficult to approach surgically and because the patients were neurologically intact, observation was the treatment of chosen. The intimal lesions were found to stabilize or improve over time with conservative therapy [45]. In a separate study investigating anticoagulation therapy, two patients out of twenty received no treatment at all for mural injuries to the internal carotid arteries. Only one of the patients survived without any neurological deficits; the other died due to severe head injury [46]. The use of anticoagulation therapy is very effective and is often used in combination with surgical or endovascular treatments. The use of heparin or aspirin has been advocated and in the study by Colella et al; there were no neurological deficits in the heparin or aspirin groups [46].

Intravenous heparin can be used in patients whose injuries are not surgically accessible and in those with delayed or fixed neurological deficits. A recommendation by Fakry et al is to complement heparin therapy with follow-up angiography, head CT's, and the patient's clinical status to determine if further treatment is necessary [47]. Patients with carotid artery intimal flaps or dissections and who are asymptomatic can be treated non-operatively with success [48]. Another study demonstrated that in patients with internal carotid dissections from blunt trauma, anticoagulation showed an improvement in 60% and no change in the clinical condition of another 23% [49]. Biffl et al found that systemic anticoagulation was associated with improved neurological outcomes in patients with and without stroke and may prevent injury progression, stroke, and worsening of neurological deficits [11]. Finally, a recent study that compared the outcomes of patients with carotid artery injury treated with anticoagulation/antiplatelet therapy versus those undergoing carotid stenting demonstrated a significantly higher rate of vessel occlusion in the stent group when compared to patients in the antithrombotic group (45% vs. 5%, respectively; p < 0.05) [50].

Overall, anticoagulation therapy is very beneficial and is associated with the lowest morbidity when compared to more invasive treatments [27,51,52].

### Surgical therapy

The second treatment group includes surgical repair of the injured vessel. Most commonly, the surgical procedures used are bypass grafting with revascularization, ligation or vessel trapping, or direct repair with oversewing (as seen in uncomplicated venous sinus injuries) [19,53-55]. The decision on which surgical procedure to employ is dependent upon the timing of symptoms, the type of injury encountered, and the location of the lesion. The timing from the onset of neurological symptoms to the initiation of therapy is crucial. When contemplating revascularization therapy, reinitiating blood flow into the area of the brain that was deprived should be accomplished within 4 hours from the onset of the neurological deficit [19]. Exceeding the four-hour time limit for revascularization may cause reperfusion insults and life-threatening intracranial edema and hemorrhage. Beyond the four-hour window, ligation of the vessel following documented adequate collateral circulation tends to be the most common surgical treatment.

The type of injury to the vessel also guides the decision of which surgical procedure to perform. For instance, aneurysms can be treated by either trapping the injured vessel or by bypass grafting. Rostomily et al reported two successfully treated cases of penetrating internal carotid artery injuries by use of bypass grafting [56]. Cervical-to-petrous internal carotid artery vein bypass grafts were used for two patients with gunshot wounds to the distal cervical/petrous internal carotid arteries. The vein bypass grafts maintained their patencies for more than 2 years with no evidence of neurological deficits [56]. The greater the urgency of the case, and the degree to which the patient is bleeding, will favor trapping of the vessel. This is often the only surgical means of treatment, since many pseudoaneurysms involve dissections that may lead to complete transections [57,58].

The location of the injured vessel not only dictates which surgical procedure to perform, but it may also determine whether a surgical treatment will be used at all. Cervical Zone II injuries are easily accessible via oblique cervical approaches, and many options for repair or ligation exist [59]. In contrast, penetrating injuries at the skull base or injuries to the cavernous sinuses from blunt trauma are not as easily accessible. It is these cases where surgery may become complicated or even dangerous that endovascular therapy via transcatheter interventional techniques may be best utilized [60].

### Endovascular therapy

Endovascular therapy often offers another therapeutic option for these patients. The use of transcatheter interventions has improved access to surgically inaccessible vessels and has reduced the need for general anesthesia as well as reduced blood loss secondary to trauma [61]. The added benefit of performing endovascular therapy during angiography has increased its use in trauma patients and is used in conjunction with both medical therapy as well as in stabilizing patients prior to definitive surgical intervention. Transcatheter interventions can be categorized into embolization, stents, and stent grafts.

Embolization therapy involves the use of agents to occlude vessels that are often actively bleeding. There are numerous embolization agents used to achieve the desired vessel occlusion; Gelfoam (Upjohn, Inc., Kalamazoo, MI), coils, and balloons are the most commonly used in neurovascular trauma. In order to decide which of these embolization materials is most advantageous for a given patient, three question must be answered. First, is the desired vessel to be embolized angiographically large or small? Second, is a permanent occlusion desired? Third, is cell death a desirable end-point [62]? If the vessel to be embolized is large, as is nearly always the case in this patient population, then larger embolic agents (Gelfoam sponge, coils) are most useful. Deciding on temporary versus permanent occlusion will dictate the use of Gelfoam versus coils or balloons. In many cases of vascular injury leading to active hemorrhage, temporary embolization is all that is needed. In such a case where temporary occlusion is desired, Gelfoam pledgets or slurry may be used to control the bleeding. Essentially, the pledgets or slurry create a cast of the vessel that is embolized, mechanically obstructing the vessel.

In the case when permanent occlusion is desired, coils may be the most appropriate embolic material to use. Embolization coils have been used to occlude traumatic fistulas, to treat acute or delayed hemorrhage from arterial lacerations, and to occlude transected or dissected carotid and vertebral arteries [63,64] (Figures 11, 12, 13). Coils, when placed in the vessel lumen, elicit a thrombogenic response by causing thrombus formation on the Dacron or nylon fibers of the coil. Thus, there is no complete mechanical obstruction until the clot forms within the fibers of the coil. This may become problematic when trauma patients who have consumed most of their clotting factors or have been transfused large volumes of packed red blood cells are unable to form a thrombus capable of causing an occlusion. In these cases, combining coils with Gelfoam may prove to be most beneficial than either embolization agent alone [64]; the coil acts as a strut for the Gelfoam to adhere to and creates a mechanical obstruction.

**Figure 10:**
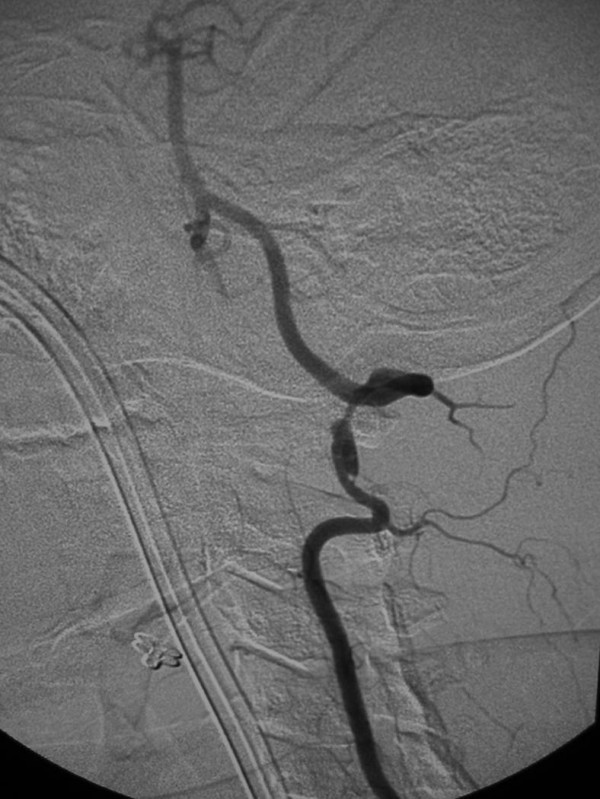
25-year old woman involved in a rollover motor vehicle collision. On admission to the hospital, a screening four-vessel angiogram revealed a pseudoaneurysm of the left vertebral artery at the level of the C1–C2 disc space. Follow-up angiography performed 7 days later revealed enlargement of the pseudoaneurysm and concomitant narrowing of the vertebral artery. Due to the tortuosity of the vertebral artery, stent placement was not deemed a viable option and the vertebral artery was embolized. Lateral digital subtraction angiogram, left vertebral artery. Notice the pseudoaneurysm arising from the distal vertebral artery (arrow), and the adjacent vertebral artery narrowing.

**Figure 11:**
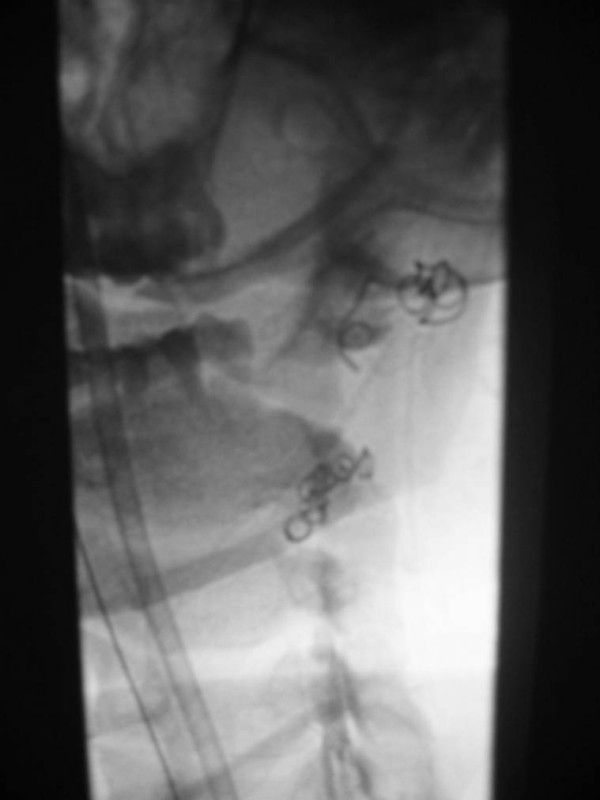
25-year old woman involved in a rollover motor vehicle collision. On admission to the hospital, a screening four-vessel angiogram revealed a pseudoaneurysm of the left vertebral artery at the level of the C1–C2 disc space. Follow-up angiography performed 7 days later revealed enlargement of the pseudoaneurysm and concomitant narrowing of the vertebral artery. Due to the tortuosity of the vertebral artery, stent placement was not deemed a viable option and the vertebral artery was embolized. Unsubtracted image demonstrating coils placed distal and proximal to the pseudoaneurysm, trapping the diseased segment of vessel.

**Figure 12:**
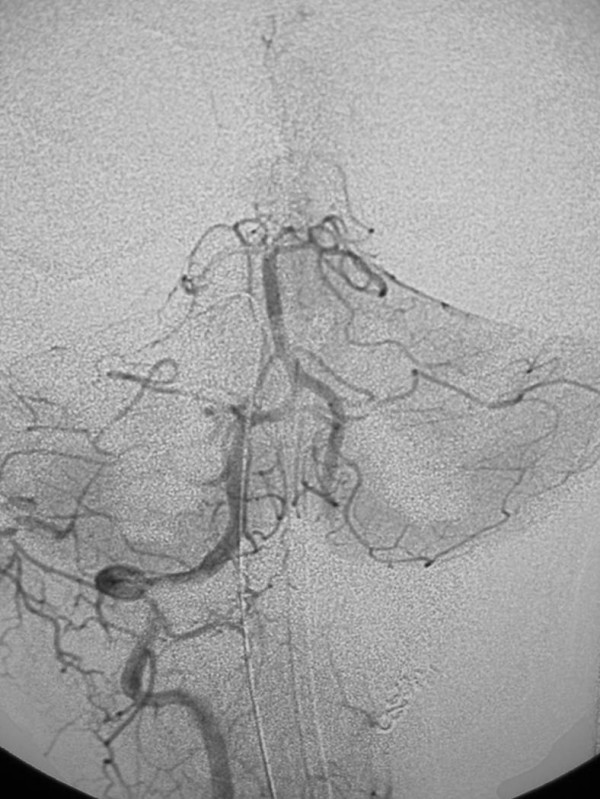
25-year old woman involved in a rollover motor vehicle collision. On admission to the hospital, a screening four-vessel angiogram revealed a pseudoaneurysm of the left vertebral artery at the level of the C1–C2 disc space. Follow-up angiography performed 7 days later revealed enlargement of the pseudoaneurysm and concomitant narrowing of the vertebral artery. Due to the tortuosity of the vertebral artery, stent placement was not deemed a viable option and the vertebral artery was embolized. Post-embolization anteroposterior digital subtraction angiogram, right vertebral artery. There is normal filling of the basilar artery, and reflux of contrast into the distal left vertebral artery which fills the left posterior inferior cerebellar artery (arrow). Images courtesy of Guido Scatorchia, MD.

Balloons may also be used for embolization of injured vessels. Brosnahan et al reported a 100% technical success rate in the treatment of eleven patients with traumatic carotid-cavernous sinus fistulas by using balloon embolization [65]. The balloon embolization technique yielded an 83% success rate in preserving the internal carotid artery; in the two cases where the internal carotid artery was embolized, there was no evidence of neurological deficits [65]. Balloon embolization may also be useful in vertebral arterial injuries to prevent further damage. Placement of the balloon proximal to the vessel lesion may reduce the incidence of fistula or false aneurysm formation as well as prevent thrombus embolization if the vessel remains patent or recanalizes [19]. Mehringer et al also demonstrated the successful use of balloon embolization for fistulae [66]. The group showed that balloons may be used to either embolize the fistula or trap the injured vessel such that surgical intervention was not required [66]. Balloons, however, are not currently commercially available in the United States.

The decision to perform embolization therapy must be made with caution, since many important collateral vessels exist between the external carotid artery and the intracranial circulation. The danger of these collateral pathways is that non-target embolization may occur. An important connection may exist between the middle meningeal artery and the ophthalmic artery. This may become crucial as the middle meningeal artery has been reported to be a common location for post-traumatic arteriovenous fistulae [67-69]. Other potential collateral pathways are between the internal carotid artery and the vertebrobasilar arterial system. The anastomoses may represent persistent embryological connections, with the trigeminal artery being the most commonly visualized. Knowledge and recognition of these potential collaterals is crucial in preventing potentially devastating nontarget embolization.

Stents are a second form of transcatheter intervention that is commonly used in trauma patients. Numerous sizes and types of stents are utilized for many different clinical applications; covered and bare stents are both used in the neurotrauma setting. Covered stents, also called stent grafts, have a benefit when used in cases involving pseudoaneurysms or arteriovenous fistulae. The disruption of the vessel wall is covered by the stent graft, and the lesion is mechanically occluded. Not only does the stent immediately resolve the vessel wall lesion, it has the added benefit of maintaining blood flow through the lumen of the vessel [70]. Thus, the injured vessel may be salvaged and the potential for ischemic events in the downstream distribution can be reduced. Scavee et al report using a covered stent to treat a traumatic pseudoaneurysm of the right internal carotid artery. They report, which obliterated the pseudoaneurysm and preserved the patency of the carotid artery even at 6-months post-procedure [71].

Bare stents also can be applied to dissection injuries and pseudoaneurysms (Figures 10, 11, 12). The bare stent is ideal in cases where the dissection is flow-limiting. The bare stent will approximate the myointimal flap with the remaining layers of the vessel wall, preventing worsening of the dissection. Also, bare stents can be used in conjunction with coils in the treatment of pseudoaneurysms. Following placement of the stent across the neck of a pseudoaneurysm, coils may be placed through the struts of the stent to pack the pseudoaneurysm. The stent traps the coils, prevent distal embolization as coils are introduced to the lesion [72]. The stent also acts to reinforce the vessel wall across the aneurysm, decreasing the chance of rupture such that coils may be packed more tightly. This also helps to prevent coil migration or dilation of the injured vessel [73].

The success of stent placement has been described in multiple small studies. Bush et al demonstrated the 100% primary technical success rate with endovascular stent placement in patients with carotid artery pseudoaneurysms [74]. The use of stents alone was successful in all five of their patients and proved to completely obliterate the pseudoaneurysms in all cases, and the endovascular stent therapy allowed the patients to avoid the morbidity associated with the surgical approach needed to access the carotid arteries at the skull base [74]. In an additional study of endovascular Palmaz stents used to treat pseudoaneurysms, Horowitz et al showed patency of the stents at 3-months follow-up [75]. They used stents on pseudoaneurysms involving the internal carotid and vertebral arteries with no evidence of refilling of the pseudoaneurysms [75].

Marks et al reported using endovascular Palmaz stents along with an 8-week course of anticoagulation on patients with cervical internal carotid artery dissections, aneurysms, and distal carotid stenosis [76]. The diameter of the injured arteries returned to normal size, and the pseudoaneurysms were approximately 90% occluded at 9 months following placement of the stents [76]. Overall, there have been satisfactory results with successful outcomes from stent placement therapy in treating internal carotid artery dissections, arteriovenous fistulas, and pseudoaneurysms [77-81].

Stent placement in the setting of trauma is not without potential complications [82,83]. In blunt trauma, often the distal aspect of a vessel, where it is fixed in position by bone, is injured. In the case of internal carotid injuries secondary to blunt trauma, the area of injury is commonly fixed at the skull base, which can pose technical challenges for stent placement. Stents that track over a 0.035-inch diameter wire are too inflexible to traverse the petrous area of the injured carotid artery. The larger delivery system also increases the risk of thrombus formation around the stent delivery system. To avoid such problems, smaller delivery systems are used with wire diameters of 0.014 to 0.018-inch. Also, stents possess the potential long-term risks of intimal hyperplasia and thrombus formation, distal migration of the stent, narrowing and restenosis of the involved vessel, and further injury to the vessel [61,75,76]. One study demonstrated a post-stent placement carotid occlusion rate of 45% in the trauma population [50]. Further comparative data of the various treatment modalities alone and in combination are required before definitive recommendations can be made for one therapy over another.

## Imaging of cervical and cerebrovascular injuries

Noninvasive imaging of cervical and intracranial vessels following traumatic injury is evolving yet remains inconsistent. Imaging studies advocated by various groups to evaluate blunt carotid and vertebral artery injury (BCVI) include ultrasound (US), computed tomographic angiography (CTA), and magnetic resonance angiography (MRA). With the recent epidemic of diagnosed BCVI, many trauma centers have pushed for an alternative to angiography – an invasive test with attendant risks. A comprehensive evaluation of these imaging modalities allows one to compare the relative risks to the accuracy of the study; only after rigorous evaluation of the data and within the limitations of an institution can an individual draw their own conclusions.

Of note, the studies to date evaluating these noninvasive imaging modalities are generally uncontrolled and noncomparative, and are often descriptive case reports and small case series. Even in those studies that compare a noninvasive imaging technique to angiography, it can be difficult to ascertain the clinical utility of the noninvasive study in question. For example, US is notoriously operator dependent, and most of the important information is gleaned from real-time examination rather than from the static images. Likewise, magnetic resonance angiography can be performed by using several different techniques, each of which could lead to greater or lesser diagnostic yields. Much of the current literature fails to discuss specific sequences used for MRA examinations; this omission makes comparison difficult, and the accuracy impossible to calculate.

An additional complicating factor in the evaluation of noninvasive imaging techniques is the rapid changes occurring in imaging technology. Over 250 articles describing MRA techniques were published – in the past 12 months alone! Significant changes in software, hardware, contrast agents, and reconstruction algorithms can potentially improve diagnostic accuracy, but confuses comparative studies. These new techniques are being described on a monthly basis – making some research obsolete shortly after the results are published.

The data presented below are grouped according to the imaging modality used, evaluating strengths, limitations, and accuracy of each modality. Distinctions are made between anatomic regions (extra-versus intracranial), as well as the mechanism of injury (blunt versus penetrating), since the accuracy of noninvasive techniques varies widely based on these two variables.

## Imaging modalities

### Duplex ultrasound (US)

Duplex US consists of gray-scale ultrasound imaging plus Doppler flow interrogation. The vast majority of currently available ultrasound units have Doppler capabilities, and the significant majority of these can also perform color Doppler examinations. Ultrasound machines are readily available in hospitals, and are currently available in many emergency and surgery departments. Ultrasound imaging provides physiologic as well as anatomic data (e.g. velocity measurements, flow direction). Secondary information can be obtained as well, such as high resistance or low velocities in the cervical carotid artery suggesting that a more remote lesion exists. Ultrasound is relatively inexpensive, with vascular ultrasound examinations of the neck costing in the neighborhood of $435 (US) (Table 2). When compared to other noninvasive or invasive examinations of the cervical vessels, US tends to require less time, and no contrast agents are required.

**Table 2:** Charges for noninvasive radiologic examinations of the cervicocerebral circulation

**Examination**	Cost*
Duplex US of the neck	$436
Transcranial Doppler	$618
CTA of the neck	$1330
CTA of the head and neck	$1374
MRA of the neck	$1374
MRA of the head and neck	$1572
Catheter angiogram of the neck (4 vessels)	$3368

*Charges (US$) at the primary author's institution as of November 2006.

There are several limitations of duplex US in the cervical region. Ultrasound examinations are operator dependent and require a highly trained and motivated ultrasonographer to adequately interrogate the entire cervical vasculature. Additionally, ultrasound may not adequately image the vertebral arteries where they enter the foramen transversaria, as well as zones I and III of the neck. Particularly when evaluating the trauma patient, direct visualization of these vessels and characterization of the injury is difficult or impossible with US; secondary evidence, such as changes in resistance or flow patterns, can suggest traumatic injury to the intracranial vessels, however these findings are inconsistent. Patients with subcutaneous emphysema in the neck cannot be examined with US due to the lack of penetration by the US waves through air-filled structures. Other superficial structures on the skin, such as overlying bandages or cervical collars, pose difficulties for US but not for other imaging modalities.

### Transcranial Doppler (TCD)

Developed in the early 1980s, transcranial Doppler is a technique that uses a low frequency, high power output ultrasound unit that allows ultrasound wave penetration through an intact adult skull. Transcranial Doppler provides a gray scale image of the brain, and can be complemented with either spectral or color Doppler to assess intracranial vessels. It can provide information in multiple clinical settings, but it is most frequently used today to detect vasospasm following intracranial hemorrhage. TCD may also be used in the detection of emboli to the intracranial circulation. Emboli, either air or particulate matter, are noted as an audible "pop" during Doppler interrogation, or as a high amplitude "blip" superimposed on the Doppler waveform [84].

The major benefit to TCD is that the procedure is completely noninvasive and can be performed at the bedside. There are many drawbacks, however, the most important of which may be the highly-operator dependent nature of the examination. TCD is a very difficult procedure to master even for a highly experienced ultrasound technologist. Once the vessel of interest is visualized, emboli are verified only by keeping the probe absolutely still for a prolonged period of time and counting the number of "blips" or "pops" noted. Such an interrogation typically will require an hour or more of constant recording, making it a procedure that oftentimes cannot be reasonably performed in a busy ultrasound practice. Although general use ultrasound machines are universally available, the special equipment required to perform this examination (low frequency transducer, high power output, recording device) is not available on all machines.

One serious limitation of TCD imaging for cervical vascular injury is that the injured vessel itself is not imaged, but rather secondary evidence of injury is obtained. The "blips" noted on TCD are highly nonspecific; the emboli can be arising from the heart, the aorta, or the cervical vessels. Indeed, it is even impossible to determine whether the embolus is an air bubble or particulate in nature, such as a thromboembolus. This nonspecificity may not limit the utility of TCD as a screening examination, but it severely limits it's utility as a true diagnostic modality.

### Computed tomographic angiography (CTA)

Computed tomographic angiography is rapidly becoming a commonly used procedure to visualize the vascular system of nearly every organ. With advancements in technology, CTA is becoming progressively faster and more accurate; current multidetector CT scanners can perform a CTA from the aortic arch to the Circle of Willis in less than ten seconds. CTA is becoming readily available, and many hospitals are equipped with the hardware and software necessary to do high-quality CTA.

CTA of the cervical vessels allows assessment of the nonvascular structures in the neck, such as the cervical spine and trachea, an added benefit that allows one to visualize nonvascular injuries. CTA not only visualizes the entire course of the cervical vessels, from their origin at the aortic arch to the circle of Willis, but also uses one bolus of contrast material, which is generally less than that used for selective angiography. Diminished contrast load is particularly important in the multitrauma patient who undergoes multiple contrast examinations. Additionally, since many patients require CT for evaluation of other injuries, adding the CTA examination at the same time saves a repeat trip to the radiology department.

In spite of the many benefits of CTA, certain drawbacks exist. Iodinated contrast material is used, and therefore CTA is relatively contraindicated in patients with renal insufficiency, diabetes, or contrast allergy. Artifacts from adjacent structures limit the utility of CTA: streak artifact from the shoulders can make assessment of zone I injuries difficult, dental fillings can obscure zone I or II injuries, and retained metallic fragments can cause severe artifact throughout the image. Image quality degradation can also occur in the thorax for patients who are unable to hold their breath.

### Magnetic resonance angiography (MRA)

**Figure 13:**
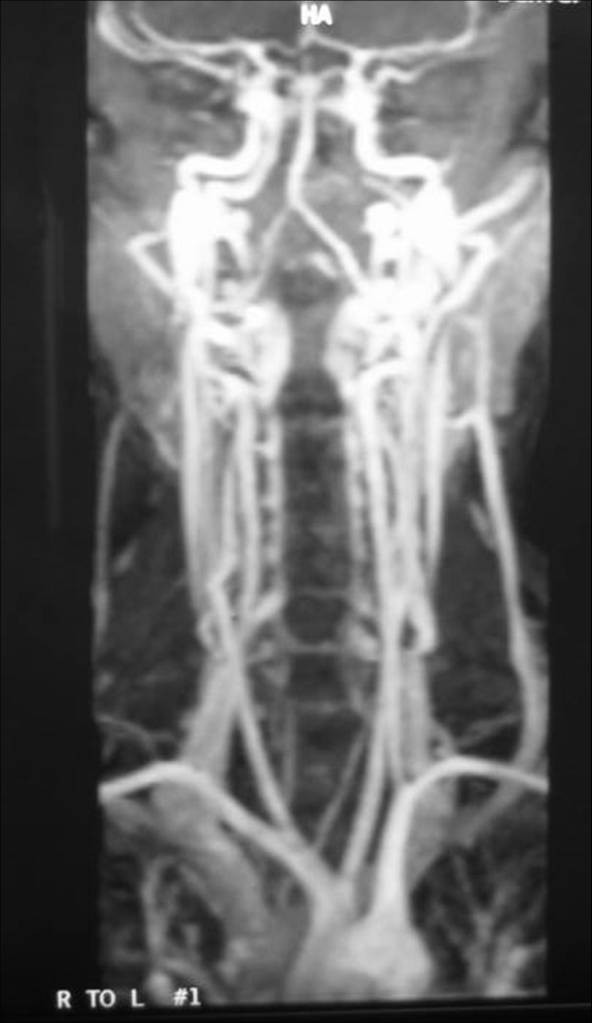
Contrast enhanced magnetic resonance angiogram demonstrating the arteries and veins of the neck. The entire arterial structures, from the aortic arch to the Circle of Willis, are demonstrated.

Similar to the technical advances seen in recent years with CTA, MRA is continually evolving. Improvements in techniques, such as faster sequence acquisitions and better contrast enhancement, have significantly improved the quality of MRA images while concomitantly allowing for faster acquisition times, thereby reducing the amount of time the patient is in the scanner (Figure 13).

MRA images can be obtained over a large area in a relatively short period of time. Although currently not as fast as multidetector CT image acquisition, an MRA with contrast enhancement can be obtained from the thoracic aorta to the circle of Willis in less than one minute. In addition, no iodinated contrast material is used. Contrast enhancement with gadolinium, quickly becoming the norm, can be used freely in patients with diabetes and renal insufficiency. Similar to CTA, MRA allows visualization of adjacent nonvascular structures, although the resolution is not as good as that seen with CT. One important potential advantage of using MRA is the ability for MR images to detect ischemic changes within the brain far sooner than that seen with any other imaging modality. Early detection of ischemic changes in the brain, coupled with a vascular injury in the same distribution, might alter therapy.

Although MRA has significant advantages, several limitations exist. Magnetic resonance scanners are not available in all hospitals, and good quality MRA images require specific software package upgrades to most of the commercially available units. Specific nonmagnetic life support systems are required in patients entering the MR unit. As is seen with CTA, metallic fragments cause significant artifact throughout the image. MRA images are highly dependent on the technique employed (e.g. the sequences used, post-acquisition processing techniques), and simple changes or omissions in the protocol will cause degradation of the reconstructed vascular image. Finally, certain limitations appear to be inherent in the interpretation of MRA images – for example, high-signal intensity intramural hematomas appear identical to adjacent high-signal flowing blood when time-of-flight sequences are used, making it difficult to diagnose such injuries [85].

Relative benefits and drawbacks for each imaging modality are presented in Table 3.

**Table 3:** Benefits and limitations of each imaging modality

**Modality**	**Direct visualization of injury**	**Evaluation of surrounding tissues**	**Evaluation of entire vascular tree**	
Duplex US	+	-	--	
Transcranial Doppler	---	---	--	
CTA	++	+++	++	
MRA	++	++	++	
Catheter angiography	+++	--	+++	
				
**Modality**	**Independent of operator**	**Readily available**	**Resolution of vessels**	**Low cost**

Duplex US	---	++	++	+++
Transcranial Doppler	---	-	--	++
CTA	++	++	++	-
MRA	+	-	+	-
Catheter angiography	-	--	+++	--

### Clinical studies

The published results for each noninvasive imaging modality vary widely, based in part on the mechanism of injury and in part on the specific techniques used for each modality (e.g. specific sequences used for MRA, spectral versus color Doppler imaging for US). This makes comparing results from one published series to another inherently problematic, and caution should be exercised. Therefore we have made an attempt to focus primarily on studies directly comparing noninvasive methods to the gold standard, catheter angiography.

The type of vascular injury identified often varies with the injury mechanism. Penetrating vascular injuries in the neck include arteriovenous fistulae, pseudoaneurysms, and vascular occlusions, while blunt mechanisms tend to cause dissections, intimal injuries, and pseudoaneurysms. When compared to intimal injuries or small dissections that may be seen with blunt traumatic injury, the injuries associated with penetrating mechanisms tend to be more readily visualized on noninvasive imaging studies.

### Duplex US

Although routinely used to diagnose and quantify atherosclerotic disease in the cervical vasculature, duplex US of the cervical vasculature in the trauma population appears to have significant limitations. It is most useful in the setting of blunt carotid or vertebral artery dissection, and in the setting of penetrating trauma to zone II (cricoid to angle of the mandible) of the neck. Multiple case reports and small case series have demonstrated the utility of duplex US in the diagnosis of dissection [86-89]. Cervical dissections can be directly visualized by gray scale ultrasound as intimal flaps and reversal of flow in the false lumen, as well as other direct or indirect US findings [86].

The true accuracy of duplex US for carotid and vertebral dissections, however, remains unknown. One published report demonstrated an accuracy of duplex US of 86% (12/14) in the evaluation of blunt carotid injuries [90]. Other confirmatory studies have not been performed directly comparing duplex US and angiography.

Duplex US has been used with success in the evaluation of penetrating neck injuries [91-94]. Typically, both Zone I (clavicle to cricoid) and Zone III (angle of the mandible to skull base) injuries are unable to be interrogated by US because of overlying structures (aerated lung and clavicle in Zone I, and mandible/skull base in Zone III); however, at least one author has demonstrated success in evaluating low zone III injuries [91]. In a series of 44 patients with penetrating cervical trauma who underwent duplex US and angiography or surgical exploration, Montalvo et al demonstrated 100% sensitivity of duplex US in detecting all serious injuries of the carotid (n = 6) and vertebral (n = 4) arteries [91]. In this study, two vertebral arteries could not be adequately interrogated with duplex US, and sonography missed one instance of reversible spasm of a carotid artery [91]. In a separate study, Corr et al demonstrated no false positive or false negative examinations in 25 patients studied with both duplex US and angiography or surgical exploration following penetrating neck trauma [92]. Fry et al also demonstrated 100% accuracy in diagnosing vascular cervical injury in 15 patients undergoing screening duplex US when compared to catheter angiography. This investigator subsequently used duplex US as a screening examination in 85 patients, reserving angiography for those individuals with positive duplex US examinations; with clinical follow-up, they reported no missed injuries using this US-driven protocol [93].

Duplex US is limited in the evaluation of intracranial vascular injuries (due to overlying osseous structures). One recent study evaluated Doppler flow velocities in the V3-segment of the vertebral arteries in patients with cranio-cervical junction (CCJ) trauma [94]. Flow velocities were noted to be abnormal in patients with CCJ, but the findings appear to lack specificity to determine the type or specific level of injury. Further studies are warranted.

### Transcranial Doppler (TCD)

Transcranial Doppler is used most frequently in the evaluation of intracranial vasospasm following intracranial hemorrhage, and is occasionally used for imaging intracranial vascular lesions. In the setting of trauma, TCD is limited to the evaluation of emboli to the brain arising from more proximal cervical vascular injuries.

Transcranial Doppler for the detection of microemboli has not been evaluated exclusively in the trauma population. Some studies' results, however, may be extrapolated to the trauma population [95-99]. In a study evaluating 28 consecutive patients with acute spontaneous internal carotid dissection, Molina et al demonstrated a significant relationship between microemboli detected with TCD and the onset of early ischemic events [97]. A separate study by Seric et al evaluated the use of TCD in patients with whiplash injury. In this study, 47 patients with clinical signs of whiplash underwent TCD within a month of their injury and at 6 months post-injury. The authors demonstrated disturbances in the intracranial flow patterns in over half of the patients; in these patients, half of the abnormalities resolved by the 6-month follow-up examination. To our knowledge, TCD has not been evaluated specifically in the evaluation of blunt cervical trauma, either as an initial screening test or as a noninvasive method to follow-up known traumatic injuries. Reports of TCD use in the trauma population are limited to scattered reports in the literature.

In one interesting case, Kilic et al used TCD to evaluate patients suspected of having a traumatic CCF [100]. The authors' conclusion was that TCD is valuable in the evaluation of these patients because of the specificity of findings in patients with CCF, and because of the relative insensitivity of other cross-sectional imaging modalities (such as CT and MRI) to diagnose this particular vascular injury. Further studies in the trauma population are needed.

### Computed tomographic angiography (CTA)

Computed tomographic angiography is perhaps the most investigated noninvasive imaging modality in the trauma population. Given it's speed, ready availability, and continued improvements in quality, CTA will likely continue to gain popularity as a screening modality for vascular trauma.

Although there have been many reports regarding the utility of CTA in the trauma patient, many do not have confirmatory angiographic or operative findings [101-106].

Computed tomographic angiography has been evaluated in the setting of penetrating trauma to the neck. Munera et al compared CTA and catheter angiography in 60 patients with penetrating neck trauma. In their study, 146 cervical vessels were evaluated, in which 10 (6.8%) arterial injuries were identified. Computed tomographic angiography correctly demonstrated nine of the 10 injured arteries, and all of the normal arteries. In this study, CTA demonstrated a sensitivity of 90%, specificity of 100%, positive predictive value of 100%, and a negative predictive value of 98% [107]. In a separate report, Gonzalez et al evaluated helical CT scanning (not dedicated CTA) in the assessment of penetrating injuries to zone II of the neck. When compared to clinical examination alone, the authors demonstrated that CT missed 50% of esophageal injuries, and 43% of internal jugular injuries. The authors concluded that helical CT has a limited role in the evaluation of patients with penetrating zone II neck injuries [108].

With the advent of multidetector CTA, identification of penetrating neck trauma may prove less problematic. Inaba et al prospectively studied the use of 16 row multidetector CT angiography as a screening tool in hemodynamically stable patients with penetrating neck injuries [109]. Over a 16 month period, 91 patients underwent multidetector CT angiography. Twelve injuries requiring operative management were identified, along with five false positives, and two indeterminate scans due to metallic artifact. Catheter angiography was not performed to confirm CTA results. There was a mean follow-up time of 33 days in 84.5% of the patients with negative studies, and none showed any signs of clinically significant neurovascular injury. The authors concluded that multidetector CTA was an effective screening tool for hemodynamically stable patients with penetrating neck injuries, but cautioned that further evaluation was warranted.

The data for CTA in the evaluation of blunt cervical vascular trauma were initially less impressive than published results for penetrating trauma. As described above, one potential cause of this discrepancy is that the more readily diagnosed injuries (e.g. pseudoaneurysms, arteriovenous fistulae, occlusions) are more frequently associated with penetrating trauma, while intraluminal abnormalities (e.g. intimal defects, dissections) are more commonly seen following blunt trauma. The latter injury types are more difficult to visualize with cross-sectional imaging techniques.

Two series compared single detector CTA and catheter angiography in the trauma population [26,110]. Biffl et al evaluated 46 patients who underwent both CTA and angiography. In their study, CTA demonstrated a sensitivity of 68%, specificity of 67%, positive predictive value of 65%, and negative predictive value of 70%. Perhaps not surprisingly, CTA was particularly insensitive in patients with grade I injuries (intraluminal irregularity <25%), missing over half (55%) of these abnormalities [110]. In a similar study, Miller et al evaluated 143 patients following blunt trauma with both CTA and catheter angiography. In their study, CTA had a sensitivity for injury to the carotid and vertebral arteries of only 47% and 53%, respectively. Although highly specific (99% for both types of injuries), CTA missed multiple injury types, including five occlusions and 11 dissections. Of clinical importance, three of nine (33%) carotid injuries missed by CTA resulted in stroke [26].

Though initial results using single detector CTA were not impressive when compared with angiography, the development of multidetector CTA has spurred new studies into the use of CTA in the detection of blunt neurovascular trauma. Berne et al evaluated 435 patients over a 22 month period who fit criteria for possible BCVI and employed 16 row multidetector CTA to detect injury [111]. Twenty-five injuries in 24 patients were identified, representing 5.5% of the screened population. All positive studies were confirmed with angiography, and no false positives were reported. Eleven of the injuries were to the vertebral artery and grade III in severity on the AAST injury scale; the remaining fourteen were carotid artery injuries, twelve of which were grade III or less in severity. The study also compared 16 row multidetector CTA with 4 row multidetector CTA and found that the former identified three times as many injuries as the latter. The authors concluded that with its ability to detect low grade injury effectively, 16 row multidetector CTA can safely be used as a screening tool in patients with suspected blunt neurovascular trauma.

Similarly, Biffl et al evaluated 331 patients with suspected BCVI over an 11 month period with 16 row multidetector CTA [112]. Eighteen patients with 20 injuries were identified. Subsequent angiography confirmed 17 of the injuries; the remaining three did not undergo angiography but were confirmed with MRA and Doppler ultrasound. Four false positives were also noted. None of the patients with negative CTA studies developed any signs of neurovascular injury. Grading of the injuries by CTA matched well with angiography as 16 of 17 injuries were graded the same. The studies by Berne and Biffl illustrate the improved ability of 16 multidetector CTA in the detection of BCVI. Neither, however, addresses its accuracy when compared with angiography since angiography was not performed in all patients who underwent CTA.

Addressing this issue, Eastman et al prospectively identified 162 patients at risk for BCVI and screened each using a 16 row multi-detector CTA [113]. Of these 162 patients, 146 subsequently underwent confirmatory angiography regardless of CTA results. Forty-five BCVIs were identified on CTA, with angiography confirming all injuries. One false negative CTA was identified on subsequent angiography in a patient with a grade I vertebral artery injury. The remaining negative CTA studies matched subsequent angiography results. The study found that the overall sensitivity, specificity, positive predictive value and negative predictive value of diagnosing BCVI with CTA were 97.7%, 100%, 100%, and 99.3% respectively. More importantly, accuracy of multidetector CTA was 99.3% when compared with angiography. The authors concluded that 16 row multidetector CTA, given its accuracy, rapidity, and non-invasiveness, is a good screening tool for patients with suspected BCVI.

Evaluation by CTA of intracranial vascular injury is limited. In particular, the paucity of literature on CTA in the evaluation of CCF suggests that the examination adds little to the CT diagnosis. Although CTA can be very useful in the evaluation of intracranial aneurysms, its role in the evaluation of intracranial vessels in the trauma population is marginal at the current time.

### Magnetic resonance angiography (MRA)

The majority of studies evaluating the utility of MRA in the trauma population are often case reports or small, noncomparative outcomes studies [110,114-122]. A distinct paucity of literature regarding MRA in patients with penetrating trauma exists. However, three studies from the recent literature directly compare MRA and catheter angiography in blunt trauma patients.

Kral et al compared 31 MRA examinations with catheter angiography in the evaluation of blunt vertebral artery injury. Of the five vascular injuries noted, MRA identified four (80% sensitivity). The authors did not report on any false-positive MRA interpretations [122].

Biffl et al reported on 16 patients undergoing both MRA and catheter angiography [110]. In their small series, the sensitivity, specificity, positive predictive value, and negative predictive values for MRA were 75%, 67%, 43%, and 89%, respectively. The authors note a high number of false-positive MRA studies (57%), suggesting MRA may often overcall lesions [110]. Similar to their results for CTA, in a study evaluating MRA in 21 patients Miller et al demonstrated low sensitivities for both carotid (50%) and vertebral artery (47%) injuries, however there was excellent specificity of MRA (97–100%) [26].

Current application of these studies are limited due to technical/procedural issues. The MRA examinations in one study were performed on a low strength magnet (0.2T) and with noncontrast time-of-flight imaging only [26]. While the other two studies did not define their MRA protocols [108,117], new techniques/protocols involving gadolinium-enhanced MRA of the cervical and intracranial vessels may yield better resolution images, thereby increasing the diagnostic yield of the examination.

## Conclusion

Noninvasive imaging for both penetrating and blunt neurovascular trauma has evolved over the past several years. Improvements in technology, coupled with a better understanding of the pathologic processes to be imaged, have allowed improvements in many of the diagnostic imaging modalities. Although the trend is improving, there remains a distinct paucity of studies in the literature that directly compare noninvasive modalities to the gold standard of catheter angiography, particularly in the arena of blunt cervical trauma. The studies evaluating penetrating trauma are much more promising, which may in part be due to the type of vascular injuries seen in this patient population subset. Based on these findings penetrating neck trauma can be reliably screened with duplex US, CTA, or MRA, with only select patients requiring catheter angiography. In patients in whom an endovascular intervention, such as embolization, is anticipated, angiography my be more beneficial as an initial diagnostic modality. The use of TCD in the evaluation of CCF is intriguing, particularly given the inherent inaccuracy with the current diagnosis by CT or MRI; more investigation is warranted in this setting.
